# Severe mental illness and quality of diabetes care: a retrospective national linked cohort study.

**DOI:** 10.23889/ijpds.v7i3.1988

**Published:** 2022-08-25

**Authors:** Katy Huxley, Rhys Davies, Catherine Foster, Chris Taylor

**Affiliations:** 1 Cardiff University

## Objectives and Approach

Free School Meals (FSM) has commonly been used as a proxy for economic disadvantage or low socio-economic background within educational research in the UK, as eligibility is based upon receipt of government welfare benefits. It is also used by government in funding decisions for schools. However, in December 2021 the Welsh Government announced that the offer of FSM will be expanded to all pupils in primary schools, meaning that this measure will no longer be available as a proxy. Our objective is therefore to considers the potential of other administrative sources of socio-economic information to fill this gap. Linking administrative education data and Census 2011 data for Wales, we undertook descriptive and regression analyses to consider how Census measures of deprivation act compared to the FSM measure in predicting attainment.

## Results

Our descriptive results show that not all pupils who are eligible for FSM are captured by the Census measures. However, our regression results indicate that socio-economic indicators from the Census – namely highest household qualification, approximated social grade and economic inactivity – all behave in a similar fashion to that of the proxy measure, FSM in association with pupil attainment.

## Conclusion

We consider results in comparison to studies using survey data, and discuss implications for the future measurement of economic disadvantage in Wales among primary school pupils.

